# Matrix Stiffness Activating YAP/TEAD1-Cyclin B1 in Nucleus Pulposus Cells Promotes Intervertebral Disc Degeneration

**DOI:** 10.14336/AD.2023.00205-1

**Published:** 2023-10-01

**Authors:** Zijie Zhou, Yinxuan Suo, Jinyu Bai, Fanguo Lin, Xiang Gao, Huajian Shan, Yichao Ni, Xiaozhong Zhou, Lei Sheng, Jun Dai

**Affiliations:** Department of Orthopedics, The Second Affiliated Hospital of Soochow University, Suzhou, Jiangsu, China.

**Keywords:** intervertebral disc degeneration, matrix stiffness, nucleus pulposus cells, proliferation, YAP/TEAD1

## Abstract

Intervertebral disc degeneration is a leading cause of disability in the elderly population. Rigid extracellular matrix is a critical pathological feature of disc degeneration, leading to aberrant nucleus pulposus cells (NPCs) proliferation. However, the underlying mechanism is unclear. Here, we hypothesize that increased matrix stiffness induces proliferation and thus degenerative phenotypes of NPCs through YAP/TEAD1 signaling pathway. We established hydrogel substrates to mimic stiffness of degenerated human nucleus pulposus tissues. RNA-sequencing identified differentially expressed genes between primary rat NPCs cultured on rigid and soft hydrogels. Dual luciferase assay and gain- and loss-function experiments evaluated the correlation between YAP/TEAD1 and Cyclin B1. Furthermore, single-cell RNA-sequencing of human NPCs was performed to identify specific cell clusters with high YAP expression. Matrix stiffness increased in severely degenerated human nucleus pulposus tissues (*p* < 0.05). Rigid substrate enhanced rat NPCs proliferation mainly through Cyclin B1, which was directly targeted and positively regulated by YAP/TEAD1. Depletion of YAP or Cyclin B1 arrested G2/M phase progression of rat NPCs and reduced fibrotic phenotypes including MMP13 and CTGF (*p* < 0.05). Fibro NPCs with high YAP expression were identified in human tissues and responsible for fibrogenesis during degeneration. Furthermore, inhibition of YAP/TEAD interaction by verteporfin suppressed cell proliferation and alleviated degeneration in the disc needle puncture model (*p* < 0.05). Our results demonstrate that elevated matrix stiffness stimulates fibro NPCs proliferation through YAP/TEAD1-Cyclin B1 axis, indicating a therapeutic target for disc degeneration.

## INTRODUCTION

Intervertebral disc degeneration (IVDD), a leading cause of disability worldwide, occurs in 40% of people under 30 years of age with the incidence increasing rapidly to 90% in populations aged over 50 years [1, 2]. Risk factors for IVDD are diverse including aging, genetics, diabetes and mechanical loading [3-5]. The intervertebral discs (IVDs) consist of jelly nucleus pulposus (NP) at the center, circumferential annulus fibrosus, and cartilage endplates integrating the IVDs into the vertebral bodies [6]. NP tissues sustain the most compression force during spinal movement [7]. Nucleus pulposus cells (NPCs), as basic functional units resident in NP tissues, are important for the synthesis of extracellular matrix (ECM) components such as aggrecan (ACAN) and type II collagen (Collagen II) to maintain the gelatinous properties of NP [8].

Rigid ECM is a critical pathological feature of disc degeneration, which greatly impacts on the cell behaviors of NPCs [9, 10]. Previous studies reported that elevated substrate stiffness rearranged the cytoskeleton of human NPCs, resulting in a change of shape from shrunken to fibrotic morphology [11]. Furthermore, softer substrate promotes cell-cell interactions as well as proteoglycan production in pig NPCs [12]. Importantly, when starting with the same cell density, number of rat NPCs on rigid hydrogel is nearly three times higher than that on soft hydrogels after seven days of culture [13]. In health conditions, NPCs are relatively few and far between in NP tissues [14]. The proliferation of NPCs is considered to be associated with disc degeneration [15]. Excessive proliferation of NPCs is likely due, in part, to rigid ECM in severely degenerated NP tissues through an unrecognized pathway. Successful treatments may thus require an in-depth understanding of the relationship between matrix stiffness and NPCs proliferation.

Higher matrix stiffness enhances Yes-associated protein (YAP)/transcriptional coactivator with PDZ-binding motif (TAZ) nuclear localization and target gene induction [16]. YAP/TAZ and TEA domain transcription factor (TEAD), as downstream effectors of the Hippo pathway, form a complex to induce cell proliferation in multiple organs [17, 18]. For example, YAP/TAZ is activated by rigid ECM and promotes proliferation and survival in epithelial cells [19, 20]. Stimulation of YAP/TEAD also enhances cancer growth and fibroblast invasion [21, 22]. Moreover, YAP depletion results in cell cycle arrest in a bone cement-induced IVDD model [23]. TEAD is an endonuclear co-transcription factor of YAP/TAZ and synergistically activates expression of various target genes [24]. YAP/TAZ-TEAD1 complex induces cell proliferation mainly through Cyclin D1, which promotes cell cycle progression at the restriction point in the G1 phase [25]. Although cell cycle has traditionally been considered the main pathway of proliferation, increasing evidence indicates that cell cycle also mediates differentiation [26]. Various cell types have been reported to be affected by cell cycle and develop into different cell types, especially stem cells [27, 28]. NPCs are divided into several subclusters depending on their specific markers through single-cell RNA-sequencing (scRNA-seq) [29], but how YAP-induced proliferation modifies the cellular components of NPCs has not been thoroughly elucidated.

In this study, we explored how matrix stiffness activated proliferation and dysregulated ECM synthesis of NPCs during IVDD. To mimic this mechanical environment of NPCs, we established polyacrylamide hydrogel substrates with varied stiffness. RNA-sequencing was used to examine the differentially expressed genes in NPCs on hydrogels and *CCNB1* was recognized as the key regulator of cell proliferation. Using a dual luciferase assay, we revealed that YAP/TEAD1 directly bound to the promoter area of *CCNB1* and enhanced its transcription. ScRNA-seq showed a high expression of YAP in fibro NPCs. In conclusion, our data demonstrated that YAP/TEAD1 activation, which was induced by increased matrix stiffness, promoted fibro NPCs proliferation by directly targeting and positively regulating Cyclin B1, resulting in fibrogenesis of NP during IVDD.

## MATERIALS AND METHODS

### Ethics statement

All animal procedures were performed according to the Institutional Animal Care and Use Committee (IACUC) of the Second Affiliated Hospital of Soochow University, Jiangsu, China. The study complied with Animal Research: Reporting in Vivo Experiments (ARRIVE) guidelines to minimize the discomfort and pain of the animals. Collection of human samples was approved by the Institutional Review Board (IRB) of the Second Affiliated Hospital of Soochow University, Jiangsu, China with informed consent forms signed by the patients (Permission number: JD-LK-2019-080-01).

### Experimental design of needle puncture model and verteporfin therapy

Eighteen male SD rats (weighing 200 g) from JOINN Laboratories (Beijing, China) were used for IVDD model establishment. All rats were anesthetized with 2% pentobarbital sodium (30 mg/kg) before surgery. The second to fourth coccygeal discs were set as Sham, Puncture and Puncture plus Verteporfin groups, respectively. In accordance with previous study [30], sham surgery was a posterolateral incision with exposure of the second coccygeal discs. Acupuncture surgery used the same approach, but exposed discs were punctured with a 21G sterile needle parallel to endplates through the ligament toward NP, and the needle was rotated at 360° and maintained for 30 seconds. A 31G sterile needle was then inserted following the same method. After acupuncture, the third coccygeal discs were injected with 0.25 ml/kg DMSO (ST2335, Beyotime, Shanghai, China) per disc and set as the Puncture group. The fourth coccygeal discs were injected with 1.25 nmol/kg verteporfin (CL 318952, Selleck, Houston, Texas) per disc in the Puncture plus Verteporfin group. Three weeks after surgery, the rats were sacrificed by CO_2_ asphyxiation and coccygeal discs were collected for histological analysis.

### Establishment of polyacrylamide hydrogel substrates

As shown in Supplementary Table 1, the concentrations of acrylamide and bis-acrylamide (79-06-1, 110-26-9, Sangon, Shanghai, China) were altered to establish polyacrylamide hydrogels of different stiffness as described previously [31]. In brief, the coverslips were incubated with 3-aminopropyltrimethoxysilane (APES, 919-30-2, Solarbio, Beijing, China), and glass slides were treated with dichlorodimethylsilane (DCDMS, 08471, Sigma-Aldrich, Burlington, Massachusetts). Acrylamide, bis-acrylamide and 0.1% TEMED (110-18-9, Sigma-Aldrich) were mixed with 1% ammonium persulfate (ST005, Beyotime) and then added onto glass slides and covered by coverslips before polymerization. Ten minutes later, the hydrogels were removed from the slides and placed into 6-well-plates for cell culture.

### Tissue preparation and mechanical characterization

Based on magnetic resonance imaging and Pfirrmann classification, NP tissues were collected from six mildly degenerated (grades II-III) and six severely degenerated (grades IV-V) human IVDs. Detailed information on the patients involved in this research is listed in Supplementary Table 2. Tissues were washed with sterile saline solution three times, embedded in optimal cutting temperature compound (BL557A, Biosharp, Hefei, China), and stored at -80°C. Tissues were cut into 40 μm slices and washed three times with PBS and then kept in a wet box at 4°C for detection.

The stiffness of human NP tissues and polyacrylamide hydrogels were measured with an atomic force microscope (AFM) scanner (Dimension ICON, Bruker, Karlsruhe, Germany) at atmospheric pressure. For measurement of the elastic modulus, a ScanAsyst-Air probe was used with a curvature radius of 50 nm and a force constant of 0.768 N/m. The modulus of NP tissues and polyacrylamide hydrogels was calculated in AFM PeakForce QNM (Quantitative Nano Mechanics) test mode after calibration of the force constant *k* and curvature radius *R* of the probes and worked out using the Hertz model in the following equation. Details can be found in previous studies [32, 33].

$$F=\frac{4}{3}\frac{E}{\left(1-u^{2}\right)}\sqrt[{}]{R}\delta^{3/2}$$

where *F* is the indentation force, *E* is Young’s modulus, *υ* is Poisson’s ratio, *R* is the radius of the indenter and *δ* is the indentation.

### Isolation, culture and treatment of rat NPCs

Male Sprague-Dawley rats (3 months old, weighing 250-300 g) from JOINN Laboratories were used for cell preparation. Rats were sacrificed by CO_2_ asphyxiation and NP tissues were dissected immediately and rinsed with saline. Primary NPCs were isolated by enzymatic digestion using 10 mg/ml Collagenase Type II (C2-BIOC, Sigma-Aldrich) for 4 hours in a shaker at 37°C. NPCs were then centrifuged at 400 ×g for 5 minutes, suspended, and cultured in Dulbecco’s modified Eagle’s medium/Ham’s F-12 (DMEM/F-12, 11320033, Gibco, Grand Island, New York) medium containing 10% fetal bovine serum (16140071, Gibco) and 1% Penicillin-Streptomycin-Amphotericin B Solution (PB180121, Procell, Wuhan, China) at 37°C, in a humidified atmosphere containing 5% CO_2_. The siRNAs to deplete *Yap* or *Ccnb1* were purchased from GenePharma (Shanghai, China) and details are listed in Supplementary Table. 3. According to the manufacturer's instructions, NPCs were seeded in 6-well plates at 70% confluency, and 100 nmol of each siRNA was transfected into cells in each well, using Lipofectamine 3000 (L3000001, Invitrogen, Waltham, Massachusetts). Otherwise, NPCs were treated with 0.25 μmol/L verteporfin or 5 μg/ml nocodazole (HY-13520, MCE, Shanghai, China) for 48 hours before collection.

### Total RNA extraction and real-time PCR (RT-PCR) determination

Total RNA was extracted from rat NPCs with TRIzol (15596026, Invitrogen). One microgram of total RNA was reverse transcribed with HiScript II Reverse Transcriptase (R201-01, Vazyme, Nanjing, China). RT-PCR was conducted with ChamQ SYBR qPCR Master Mix (Q311-02/03, Vazyme) according to the manufacturer's instructions. The primers for all genes are supplied in Supplementary Table 4.

### RNA-Sequencing analysis and resource of human NPCs scRNA-seq data

Total RNA was quantified using Qubit® 3.0 Fluorometer (Life Technologies, Grand Island, New York), and assessed for quality using the Agilent 2100 Bioanalyzer (Santa Clara, California). RNA-sequencing was performed using an Illumina Genome Analyzer, resulting in an average of 47-108 million mapped reads per sample. The raw data were subjected to QC analyses using FastQC v0.11.7 software. The transcripts were normalized to those of the control group and transcripts with low variance (< 0.1) across samples were removed. Statistically significant changes in gene expression were calculated using the DESeq2-package[34].

The scRNA-seq data were downloaded from the deposited dataset (GSE165722). We excluded cells with fewer than 500 detected genes. Clusters were named and marker genes for each cluster were identified as described in Tu et al., 2021. The data shown in Fig. 6G represent the mean expression (exp.) of genes and percentage of cells expressing the corresponding genes (pct. exp.) across all cells with a given cluster label (e.g., fibro NPCs-I).

### Western blot analysis

Cell lysates were prepared using RIPA lysis butter (P0013B, Beyotime), and the protein concentration was calculated with a BCA Protein Assay Kit (P0012, Beyotime). Protein samples were separated by SDS-PAGE electrophoresis and then electro-transferred onto PVDF membranes (1620184, Bio-Rad, Hercules, California). Five percent skim milk was used to block the membranes in TBST for 2 hours before incubating with primary antibodies including Cyclin B1 (1:1000, 12231S, CST, Danvers, Massachusetts), Cyclin D1 (1:1000, 55506S, CST), Cyclin Dependent Kinase 1 (CDK1, 1:1000, 77055S, CST), YAP (1:1000, 14074S, CST), p-YAP (1:1000, 13008S, CST), TAZ (1:1000, 83669S, CST), TEAD1 (1:500, sc-376113, Santa Cruz, Santa Cruz, California), Aggrecan (1:1000, 13880-1-AP, Proteintech, Chicago, Illinois), Collagen Type II (1:1000, 28459-1-AP, Proteintech), Connective Tissue Growth Factor (CTGF, 1:500, sc-365970, Santa Cruz) and Matrix Metallopeptidase 13 (MMP13, 1:1000, 18165-1-AP, Proteintech) at 4°C overnight. Membranes were incubated with secondary antibodies (1:1000, SA00001-2, Proteintech) at room temperature for 1 hour. After three washes with TBST, immunolabeling was activated by an Omni-ECL™ Femto Light Chemiluminescence Kit (SQ201, Epizyme, Shanghai, China) and captured by a GeneGnome XRQ analyzer (Syngen, Cambridge, UK). Gray value was analyzed using ImageJ normalized to GAPDH.

### Dual luciferase assay

HEK 293T cells, as tool cells, were seeded in 12-well plates at a density of 2×10^5^ cells per well before transfection. In each well, 1 μg of *CCNB1*-promoter-luciferase plasmid (GenePharma), 1 μg of *TEAD1* plasmid (GenePharma) and 0.1 μg of plasmid containing the *Renilla reniformis* luciferase gene (GenePharma) were transfected into cells with Lipo3000. Details are listed in Supplementary Table 3. Cells were incubated for 48 hours before harvesting for the dual luciferase assay. Luciferase activities were processed using a Dual-Luciferase Reporter Assay System kit (E1910, Promega, Madison, Wisconsin) according to the manufacturer’s instructions and detected with an Infinite 200 PRO plate reader (Tecan, Männedorf, Switzerland).

### Hematoxylin and eosin staining (HE), alcian blue and nuclear fast red staining (ABNFR) and immunofluorescence staining

Rat coccygeal IVDs were decalcified for 14 days after fixation in 4% (v/v) formaldehyde. Human NP tissues and rat discs were paraffin-embedded and sliced into 5-μm-thick sections for HE staining, ABNFR staining and fluorescence staining. Morphological changes in discs were observed under a light microscope and scored according to the classification criteria described in Supplementary Table 5.

For immunofluorescence staining, antigen retrieval was performed in Tris-EDTA buffer (pH 9.0) (ab93684, Abcam, Cambridge, UK) for 20 minutes, and then, paraffin slices were permeabilized with 0.3% (v/v) Triton-X100 (9002-93-1, Sangon) for 10 minutes and blocked with 10% (v/v) goat serum (C0265, Beyotime) for 1 hour. Primary antibodies against Cyclin B1 (1:100, ab181593, Abcam), YAP (1:100, ab205270, Abcam) and Collagen Type II (1:100, 28459-1-AP, Proteintech) were incubated overnight at 4°C. Slides were washed with PBST three times and incubated with secondary antibodies (A-11004, Invitrogen, Waltham, Massachusetts) for 1 to 2 hours. Nuclei were stained with DAPI (62248, Thermo Fisher) for 5 minutes. The images were captured with a confocal laser scanning microscope (LSM800, Zeiss, Oberkochen, Germany). All sections were observed under a confocal laser scanning microscope (LSM800, Zeiss) to exclude endogenous tissue fluorescence. The secondary antibody only controls were stained following the same method except that primary antibodies were replaced with PBST to distinguish genuine target staining from background. The images are selected based on results obtained from quantitative analysis and represent the mean values of data.

### Cell proliferation assay

Cell proliferation was detected using an Ethynyldeoxyuridine (EdU) detection kit (C10310-1, RiboBio, Guangzhou, China) following the manufacturer’s instructions. NPCs were cultured with EdU for 4 hours and stained with Apollo 567 and DAPI. EdU incorporation was assessed by fluorescence staining. More than 200 cells were counted for data analysis in each group.

### Flow cytometric and cell cycle analysis

NPCs were collected with 0.25% Trypsin-EDTA Solution (C0201, Beyotime), fixed in ice-cold 70% ethanol for 2 hours at 4°C and spun down (200 ×g for 10 minutes). The cell pellet was then resuspended in propidium iodide staining solution (5 ng/ml in PBS with RNase A) and incubated at 37 °C for 30 minutes according to the manufacturer's instructions (C1052, Beyotime). Flow-cytometry analyses were performed on a Beckman CytoFLEX analyzer (Brea, California).

**Figure 1.**
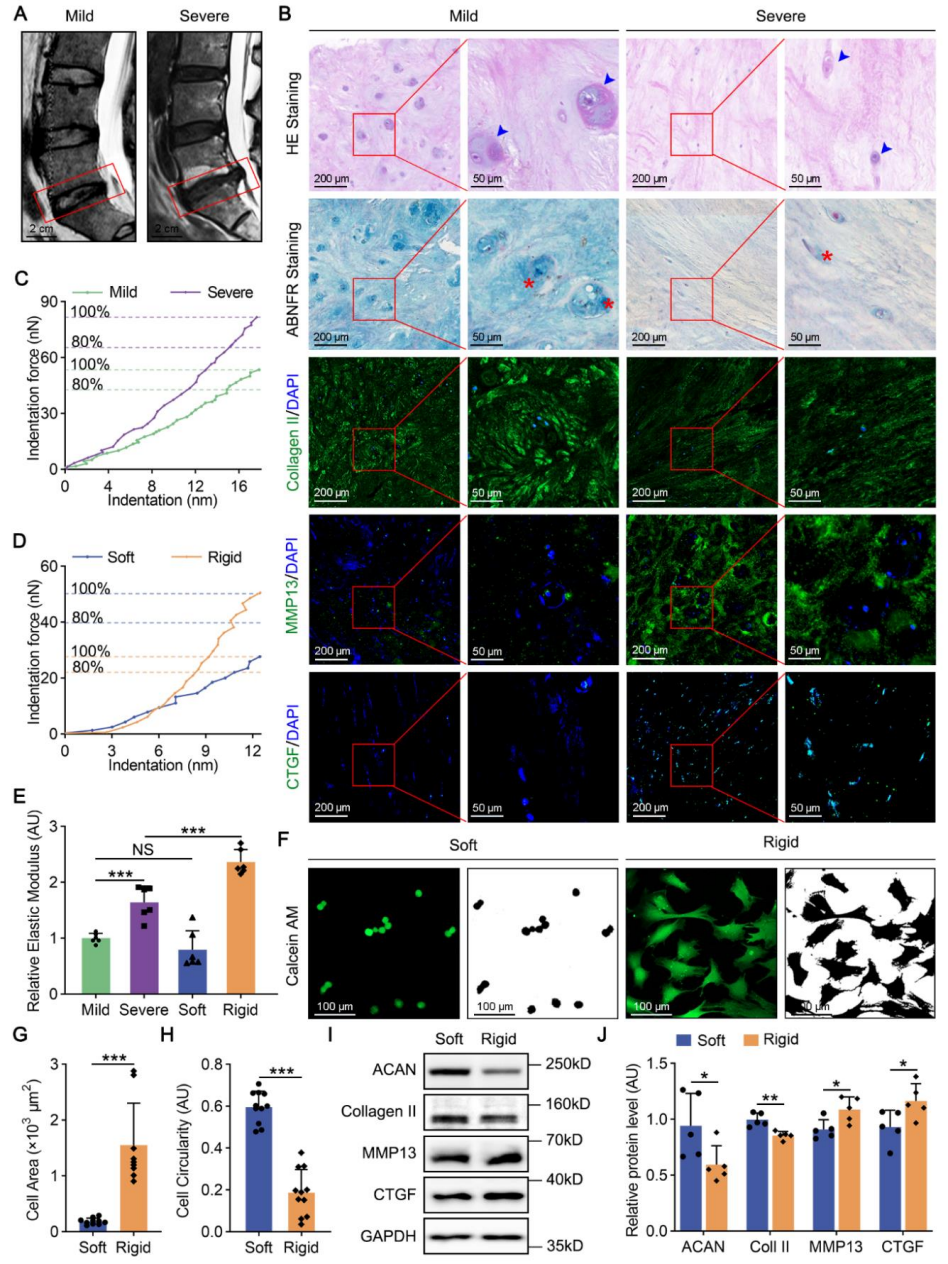
Matrix stiffness of NP tissues is increased on degenerated discs. (A) T2-Weighted MRI of human mildly degenerated (left panel) and severely degenerated (right panel) IVDs. The degenerated discs are indicated by red rectangles. (B) HE, ABNFR and fluorescence staining (Collagen II, MMP13 and CTGF) of NP tissues from human degenerated IVDs. Human NPCs (blue arrowheads) and proteoglycan (red asterisk) were labeled. (C-E) Force against indentation in human degenerated NP tissues and hydrogels. The relative elastic modulus was quantified relative to the mild group (n=6). *p*-value was derived from One-way ANOVA followed by Tukey post hoc test. (F-H) Cell area (n=10) and cell circularity analysis (n=10) of rat NPCs through Calcein AM staining. *p*-value was derived from two-tailed unpaired Student’s t-test. I-J) Western blot analysis of ACAN (n=5), Collagen II (n=5), MMP13 (n=5) and CTGF (n=5) in rat NPCs on soft and rigid hydrogels. *p*-value was derived from Wilcoxon rank-sum test. GAPDH was used as the loading control and results were relative to the soft group. Data are presented as the mean ± SD. ^*^p < 0.05, ^**^p < 0.01, ^***^p < 0.001.

### Calcein Acetoxymethyl Ester (Calcein AM) staining and analysis of cell morphology

For morphological analysis, cells were stained with Calcein AM according to the manufacturer's instructions (C2012, Beyotime). Briefly, rat NPCs were washed with PBS and incubated with 1 μmol/L Calcein AM at 37°C for 60 minutes. Images were captured by a confocal laser scanning microscope (LSM800, Zeiss). Cell circularity is defined as C =4 * π (A/P^2^) (A is cell area and P is cell perimeter). The value of circularity is 1.0, reflecting a perfect circle, whereas the cell circularity is 0.0, indicating an irregular polygon.

### Disc Height Index (DHI) and related measurements

Disc height and vertebral body height were calculated according to the micro-CT results and averaged per specimen. DHI was calculated as previously described [35] and determined by the following equation:

$$\mathrm{DHI}=\frac{2x(\mathrm{DH}1+\mathrm{DH}2+\mathrm{DH}3)}{A1+A2+A3+B1+B2+B3}$$

A and B represent the height of the vertebral bone immediately rostral and caudal to the IVD of interest, respectively. DH represents the disc height between the two adjacent vertebrae (A and B).

### Statistical analysis

The Shapiro-Wilk test was performed to evaluate the normality of the numerical data. Normally distributed data are presented as the mean ± standard deviation (SD), while non-normally distributed data are presented as the median (25th percentile, 75th percentile). A two-tailed unpaired Student’s *t*-test was used for comparisons between 2 groups obeying normal distribution. One-way ANOVA followed by Tukey post hoc test was applied for comparisons among several groups obeying normal distribution. In condition of an abnormal distribution or a small sample size (n<6), Wilcoxon rank-sum test was applied for comparisons between 2 groups and Kruskal-Wallis test was applied for comparisons among several groups. A *p*-value of < 0.05 was considered statistically significant. GraphPad Prism 9.0 software was used for all statistical analyses.

## RESULTS

### Matrix stiffness of NP tissues is increased on degenerated discs

To identify pathological changes in degenerated NP tissues, we collected human IVDs with different grades of degeneration (mild and severe). The IVDs demonstrated different signal intensities in T2-Weighted Magnetic Resonance Imaging (MRI) (Fig. 1A). Human NPCs of severely degenerated tissues showed higher ellipticity and fewer intracellular vacuoles than cells in the mild group through HE staining. Using ABNFR and fluorescence staining, we found a dramatic reduction in proteoglycan and Collagen II in the severe group (Fig. 1B). Fibrotic anabolism, including MMP13 (an indicator of ECM degradation [36]) and CTGF (a direct YAP transcriptional target for tissue fibrosis [37]), was increased with more fibroblast in tissues of severe degeneration (Fig. 1B). The elastic modulus of severely degenerated tissues was approximately 65% higher than that of the mild group (*p* < 0.001, Fig. 1C, E). These data suggest that human IVDD is characterized by fibrotic morphology, degraded ECM components and increased matrix stiffness.

To mimic the mechanical environment in degenerated NP tissues, we established polyacrylamide hydrogel substrates (soft and rigid) for two-dimensional culture of rat NPCs (Supplementary Fig. 1). Soft hydrogel and mildly degenerated tissues had the same stiffness (*p* = 0.182). Rigid hydrogel was stiffer than the severe group, simulating more degenerated ECM (*p* < 0.001, Fig. 1D, E). Like human NPCs in the severe group, rat NPCs cultured on rigid hydrogel showed an elliptic and vacuole-free morphology (Supplementary Fig. 1B). Through Calcein AM staining, which examined esterase activity to exclude interference of dead cells on morphology, we found a seven-fold increase in cell area and a 30% decrease in cell circularity of NPCs on rigid hydrogel compared with those of the soft group (*p* < 0.001 for cell area, *p* < 0.001 for cell circularity, Fig. 1F-H). Notably, western blot analysis proved that rigid hydrogel reduced chondroid elements (ACAN and Collagen II) and enhanced fibrotic anabolism (MMP13 and CTGF) in rat NPCs (*p* < 0.05 for ACAN,*p* < 0.01 for Collagen II, *p* < 0.05 for MMP13, *p* < 0.05 for CTGF, Fig. 1I, J). Taken together, these results prove that increased matrix stiffness results in degenerative phenotypes of NPCs during IVDD.

**Figure 2.**
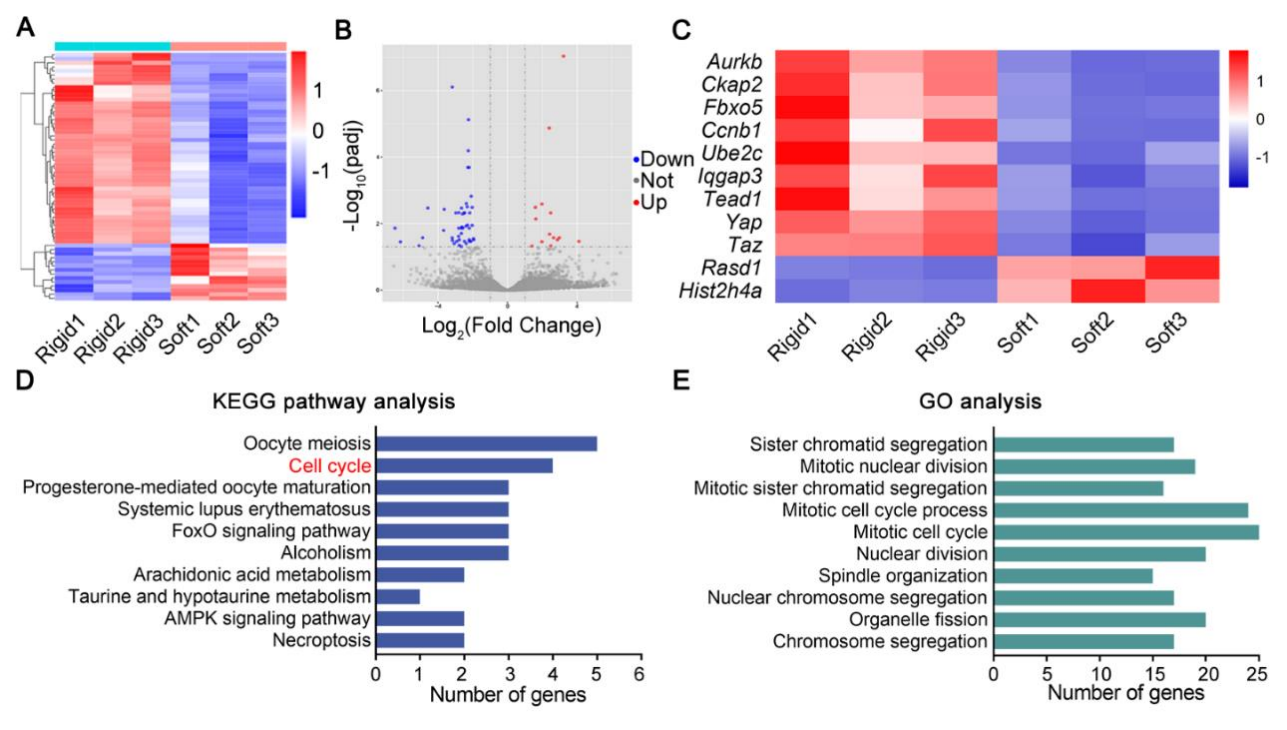
RNA-sequencing verifies activated NPCs proliferation and YAP signaling pathway on rigid hydrogel. (A) Heatmap of the RNA-sequencing data showing DEGs in rat NPCs on soft and rigid hydrogels. (B) Volcano plot showing DEGs between rat NPCs on soft and rigid hydrogels. (C) Heatmap representing cell cycle- and YAP-related genes in rat NPCs on soft and rigid hydrogels. (D, E) KEGG pathway analysis and GO analysis of DEGs in rat NPCs on soft and rigid hydrogels. The top 10 results were reserved.

### RNA-sequencing verifies activated NPCs proliferation and YAP signaling pathway on rigid hydrogel

To investigate how elevated matrix stiffness impacts on rat NPCs at the genetic level, we performed RNA-sequencing. We identified 61 significantly differentially expressed genes (DEGs), 14 downregulated and 47 upregulated, between the soft and rigid groups (Fig. 2A, B; Supplementary Table 6). Among the upregulated genes, four genes (*Yap, Taz, Tead1* and*Iqgap3*) regulate the Hippo signaling pathway and one gene (*Ccnb1*) directly controls G2/M phase checkpoint in cell cycle [38, 39]. Moreover, the expression of 6 genes (*Aurkb, Ckap2, Fbxo5, Rasd1, Hist2h4a* and*Ube2c*) regulating mototic progression was dysregulated on rigid hydrogel (Fig. 2C). Kyoto Encyclopedia of Genes and Genomes (KEGG) analysis revealed significant enrichment for genes involved in cell cycle (Fig. 2D). Gene Ontology cluster (GO) analysis showed that DEGs were enriched in the subset of biological processes with elevated levels in cell mitosis and cell cycle (Fig. 2E). Together, these results demonstrate the activation of cell proliferation and the YAP signaling pathway in rat NPCs on rigid hydrogel.

### Higher matrix stiffness promotes NPCs proliferation through Cyclin B1

**Figure 3.**
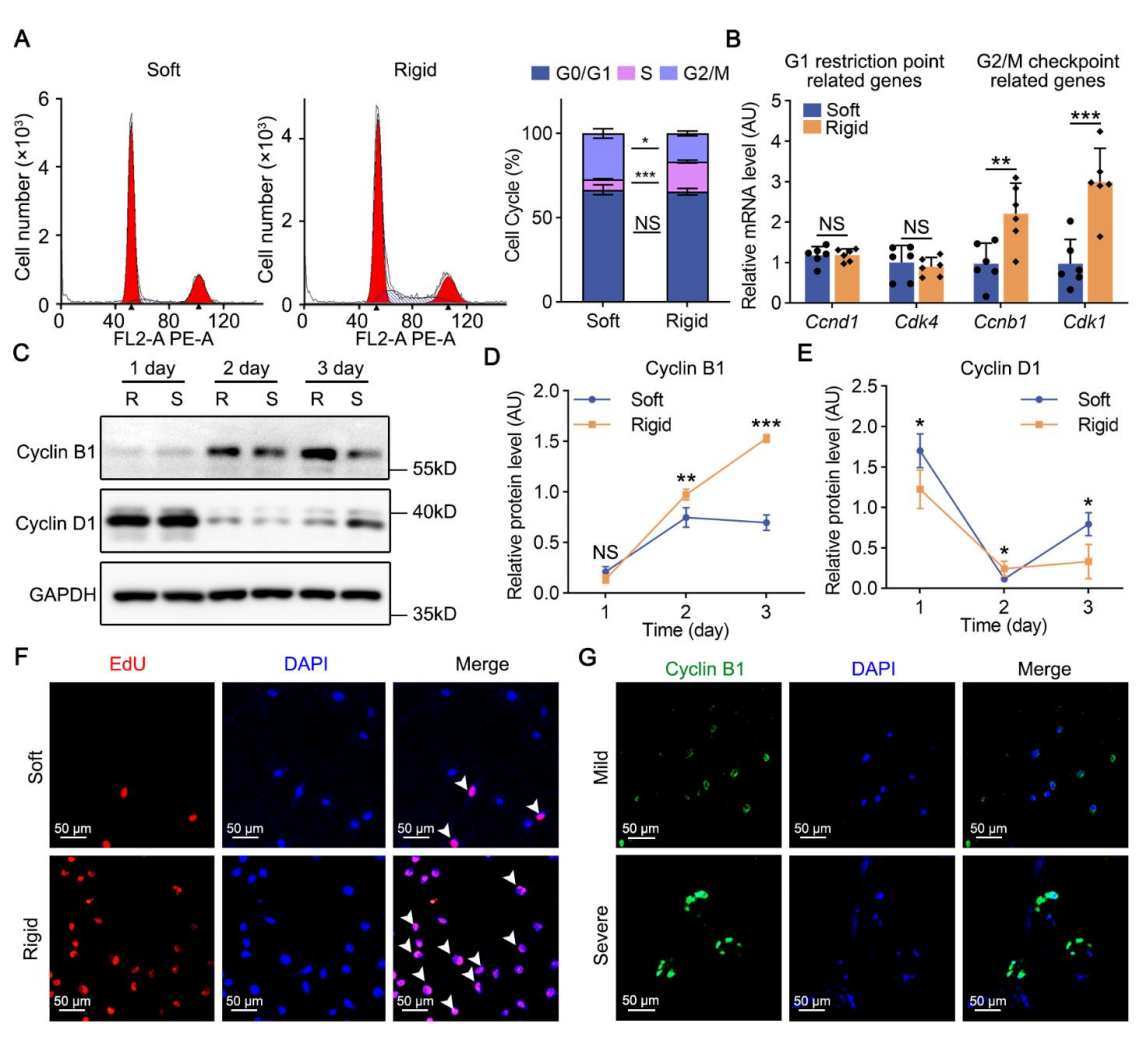
Higher matrix stiffness promotes NPCs proliferation through Cyclin B1. (A) Cell cycle analysis of rat NPCs on soft and rigid hydrogels (n=3).*p*-value was derived from Wilcoxon rank-sum test. (B) Relative mRNA levels of *Ccnd1* (n=6), *Cdk4* (n=6), *Ccnb1* (n=6) and *Cdk1* (n=6) in rat NPCs on soft and rigid hydrogels by RT-PCR. *p*-value was derived from two-tailed unpaired Student’s t-test. *Gapdh* was used as the loading control and results were relative to the soft group. (C-E) Western blot analysis of Cyclin B1 (n=6) and Cyclin D1 (n=6) in rat NPCs planted on soft and rigid hydrogels for 1, 2 or 3 days. *p*-value was derived from two-tailed unpaired Student’s t-test. GAPDH was used as the loading control and results were relative to the soft group. (F) Cell proliferation capacity analysis of rat NPCs planted on soft and rigid hydrogels for 2 days through EdU staining (n=4).*p*-value was derived from Wilcoxon rank-sum test. Proliferative NPCs are labeled with white arrowheads. (G) Fluorescence staining analysis of Cyclin B1 expression in human NP tissues with different grades of degeneration. Nuclei were stained with DAPI. Data are presented as the mean ± SD values. ^*^p < 0.05, ^**^p < 0.01, ^***^p < 0.001.

We further assessed changes in cell cycle phases through flow-cytometry. An increased proportion of cells in S phase and a decreased proportion of cells in G2/M phase were observed on rigid hydrogel, with no difference in G0/G1 phase compared with that of the soft group (*p* < 0.001 for S phase, *p* < 0.05 for G2/M phase, *p* = 0.764 for G0/G1 phase, Fig. 3A). Cyclins and CDKs are critical mediators of cell cycle progression [40]. Compared with cells in the soft group, rat NPCs on rigid hydrogel showed higher mRNA levels of *Ccnb1* and *Cdk1*, with no difference in *Ccnd1* and *Cdk4* (*p* < 0.001 for *Ccnb1*, *p* < 0.05 for *Cdk1*, *p* = 0.154 for *Ccnd1*, *p* = 0.617 for *Cdk4*, Fig. 3B). Since Cyclins are dynamic during cell cycle progression [41], we further examined their protein content on Days 1, 2 and 3 through western blot. Starting from Day 2, rat NPCs on rigid hydrogel expressed more Cyclin B1 than cells on soft hydrogel, with increases of 30.3% and 120.0% on Days 2 and 3, respectively (*p* < 0.01 for Day 2, *p* < 0.001 for Day 3, Fig. 3C, D). Cyclin D1 expression on rigid hydrogel was 28.0% and 58.5% lower than that of the soft group on Days 1 and 3, respectively, but slightly higher on Day 2 (*p* < 0.05 for Day 1, *p* < 0.05 for Day 3, *p* < 0.05 for Day 2, Fig. 3C, E). Furthermore, EdU incorporation analysis showed a striking increase in proliferation incidence on rigid hydrogel (*p* < 0.001, Fig. 3F; Supplementary Fig. 2A). Consistent with the *in vitro* results, expression of Cyclin B1 increased in severely degenerated NP tissues (Fig. 3G). In summary, higher matrix stiffness enhances cell proliferation mainly through Cyclin B1 in NPCs.

### YAP knockdown attenuates NPCs proliferation and fibrotic anabolism

**Figure 4.**
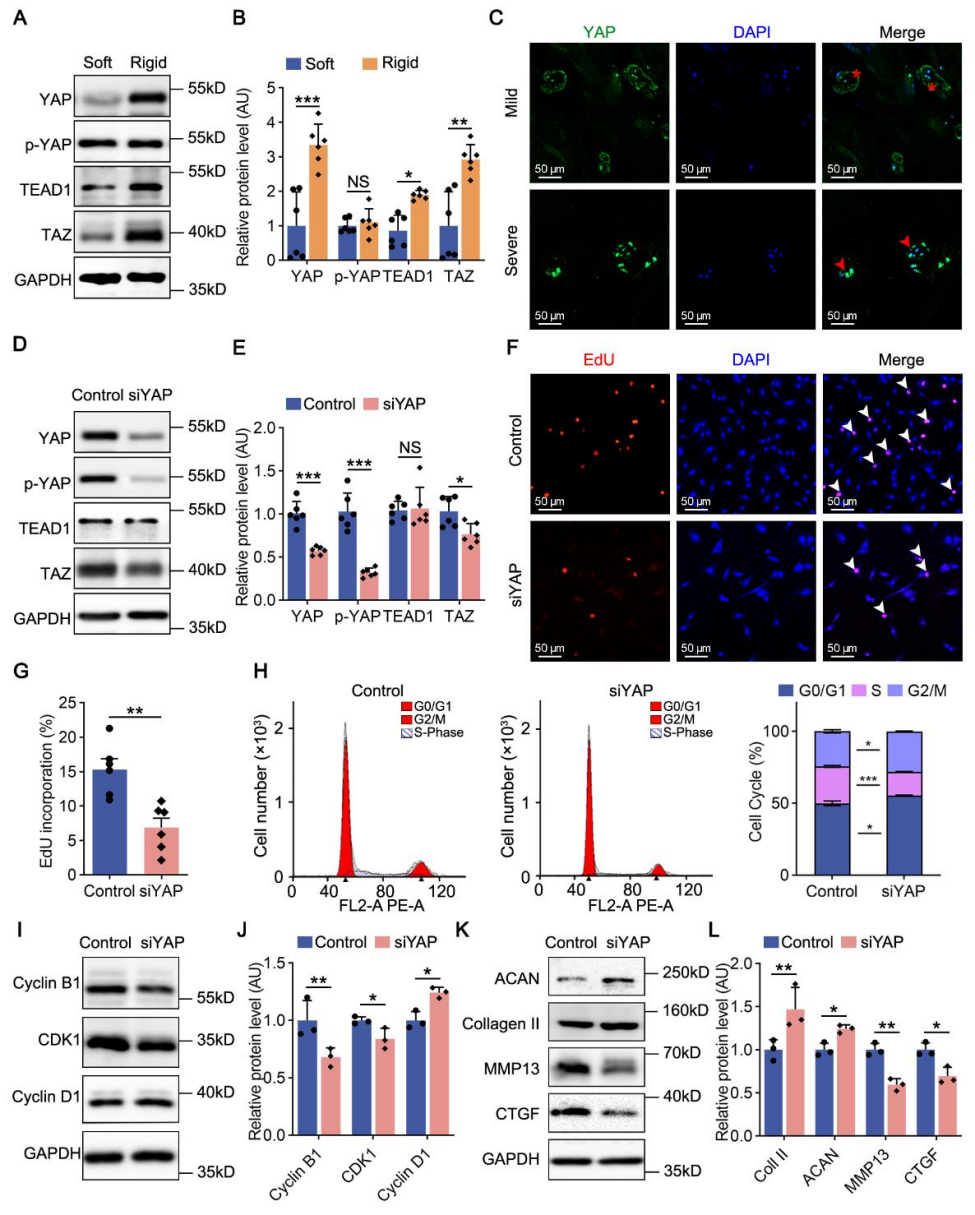
YAP knockdown attenuates NPCs proliferation and fibrotic anabolism. (A, B) Western blot analysis of YAP (n=6), p-YAP (n=6), TEAD1 (n=6) and TAZ (n=6) in rat NPCs cultured on soft and rigid hydrogels for 2 days. *p*-value was derived from two-tailed unpaired Student’s t-test. GAPDH was used as the loading control and results were relative to the control group. (C) Fluorescence staining analysis of YAP expression in human NP tissues with different grades of degeneration. Nuclei were stained with DAPI. Extranuclear (red asterisk) and endonuclear YAP (red arrowheads) were labeled. (D, E) Western blot analysis of YAP (n=6), p-YAP (n=6), TEAD1 (n=6) and TAZ (n=6) in rat NPCs transfected with siNC or siYAP (both were planted on rigid hydrogel) for 2 days. *p*-value was derived from two-tailed unpaired Student’s t-test. GAPDH was used as the loading control and results were relative to the control group. (F, G) Cell proliferation capacity analysis on rat NPCs treated as in D through EdU staining (n=6).*p*-value was derived from two-tailed unpaired Student’s t-test. Proliferative NPCs were labeled with white arrowheads. (H) Cell cycle analysis of rat NPCs treated as in D (n=3). (I-L) Western blot analysis of Cyclin B1 (n=3), CDK1 (n=3), Cyclin D1 (n=3), ACAN (n=3), Collagen II (n=3), MMP13 (n=3) and CTGF (n=3) in rat NPCs treated as in D.*p*-value was derived from Wilcoxon rank-sum test. GAPDH was used as the loading control and results were relative to the control group. Data are presented as mean ± SD values. ^*^p < 0.05, ^**^p < 0.01, ^***^p < 0.001.

Rat NPCs cultured on rigid hydrogel for 2 days showed higher protein levels of YAP, TEAD1 and TAZ than those of the soft group, indicating significant activation of the YAP signaling pathway in rat NPCs (*p* < 0.001 for YAP, *p* < 0.05 for TEAD1, *p* < 0.01 for TAZ). No difference was detected in the expression p-YAP, which is the inactive form of YAP (*p* = 0.582, Fig. 4A, B). Increased mRNA levels of *Yap*, *Tead1* and *Taz* were verified through RT-PCR (*p* < 0.05 for *Yap*, *p* < 0.05 for *Tead1*, *p* < 0.001 for *Taz*, Supplementary Fig. 2B). Moreover, fluorescence staining showed increased expression and nuclear localization of YAP in severely degenerated human NP tissues, verifying the activated YAP signaling pathway in human NPCs (Fig. 4C).

**Figure 5.**
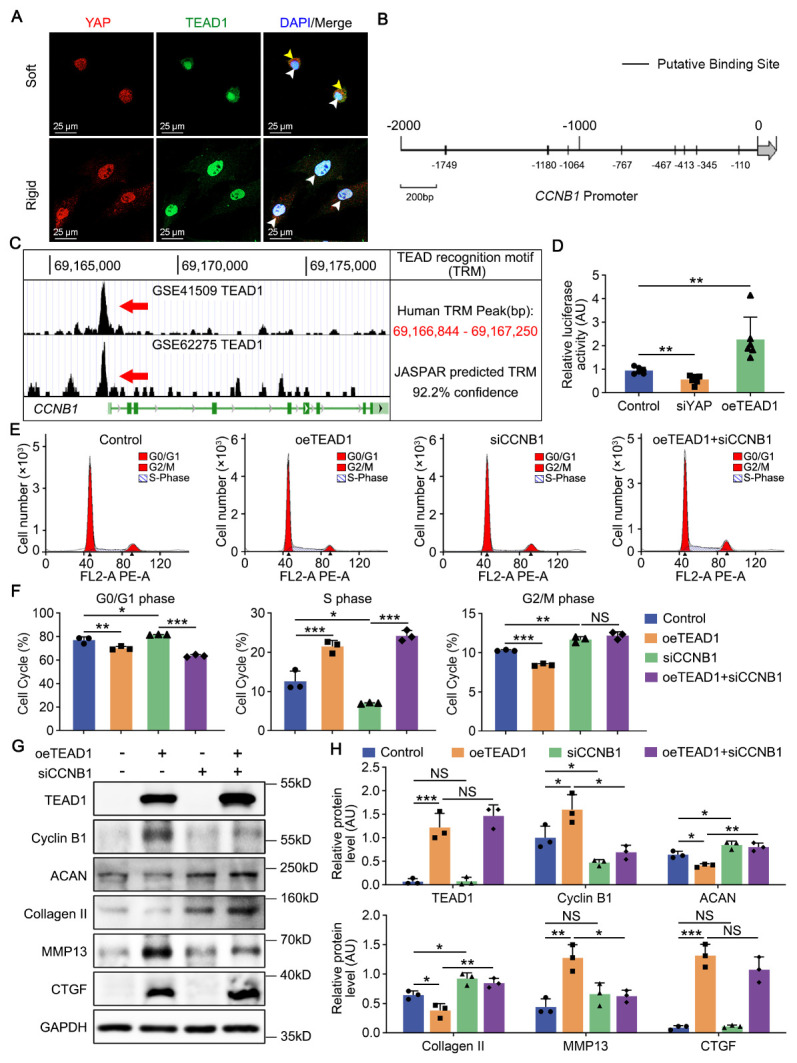
YAP/TEAD1 targets Cyclin B1 to induce changes in cell cycle and anabolic phenotypes. (A) Fluorescence staining analysis of YAP and TEAD1 expression in rat NPCs planted on soft and rigid hydrogels for 2 days. Nuclei were stained with DAPI. Separated YAP in cytoplasm (yellow arrowheads) and colocalized YAP/TEAD1 in nuclei (white arrowheads) were labeled. (B) Eight putative TRMs identified in the *CCNB1* promoter region using JASPAR database. (C) Putative TRM identified in *CCNB1* promoter region using two deposited ChIP-sequencing datasets. (D) Relative luciferase activity in rat NPCs transfected with siYAP or oeTEAD1 for 2 days examined by dual luciferase assays (n=6).*p*-value was derived from two-tailed unpaired Student’s t-test. Data were relative to the control group. (E, F) Cell cycle analysis of rat NPCs transfected with siYAP (n=3), oeTEAD1 (n=3) or both (n=3) for 2 days.*p*-value was derived from Kruskal-Wallis test. (G, H) Western blot analysis of TEAD1 (n=3), Cyclin B1 (n=3), ACAN (n=3), Collagen II (n=3), MMP13 (n=3) and CTGF (n=3) in rat NPCs treated as in E.*p*-value was derived from Kruskal-Wallis test. GAPDH was used as the loading control and results were relative to the control group. Data are presented as the mean ± SD values. ^*^p < 0.05, ^**^p < 0.01, ^***^p < 0.001.

**Figure 6.**
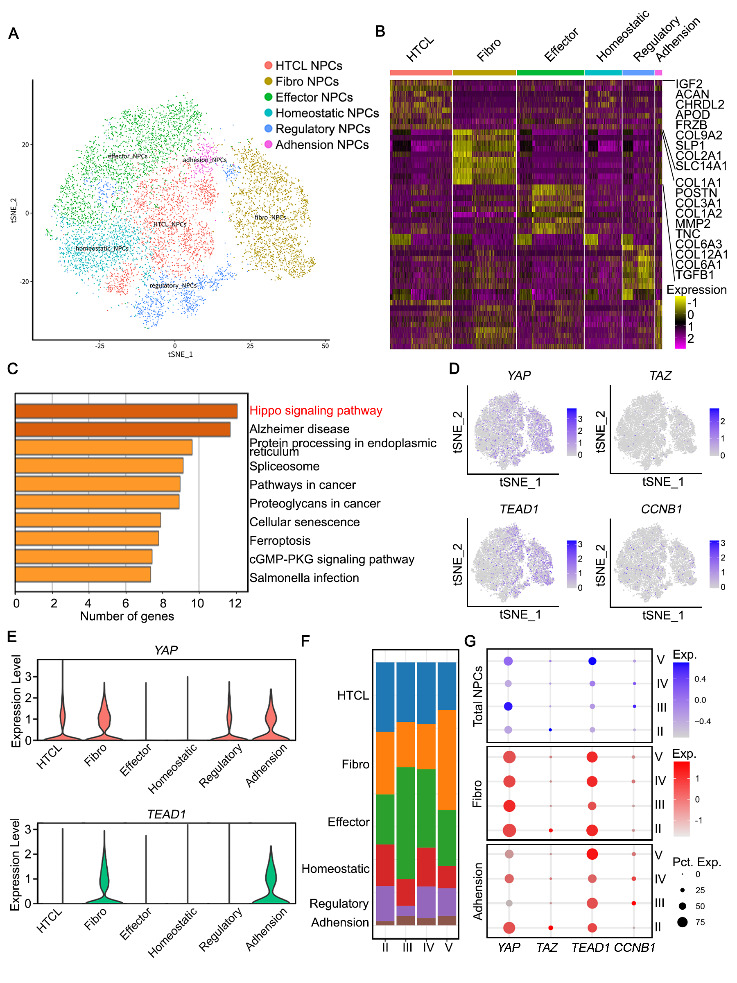
Fibro NPCs with high YAP expression enhance fibrogenesis in degenerated discs. (A) T-Distributed Stochastic Neighbor Embedding (tSNE) visualization of human NPCs identifying six different clusters after unsupervised clustering. (B) Heatmap showing the typically expressed genes in each cell cluster. (C) GO analysis of DEGs in human NPCs from severely versus mildly degenerated NP tissues. (D) Cell populations identified by shown genes. (E) Violin plots showing the expression levels of YAP and TEAD1 in each cell cluster. F) Proportion of each cell cluster in NP tissues with different Pfirrmann grades. (G) Dot plots showing expression (exp.) of *YAP*, *TAZ*, *TEAD1* and *CCNB1* in human NPCs. Dot size represents the percentage of cells expressing the corresponding genes (pct. exp).

**Figure 7.**
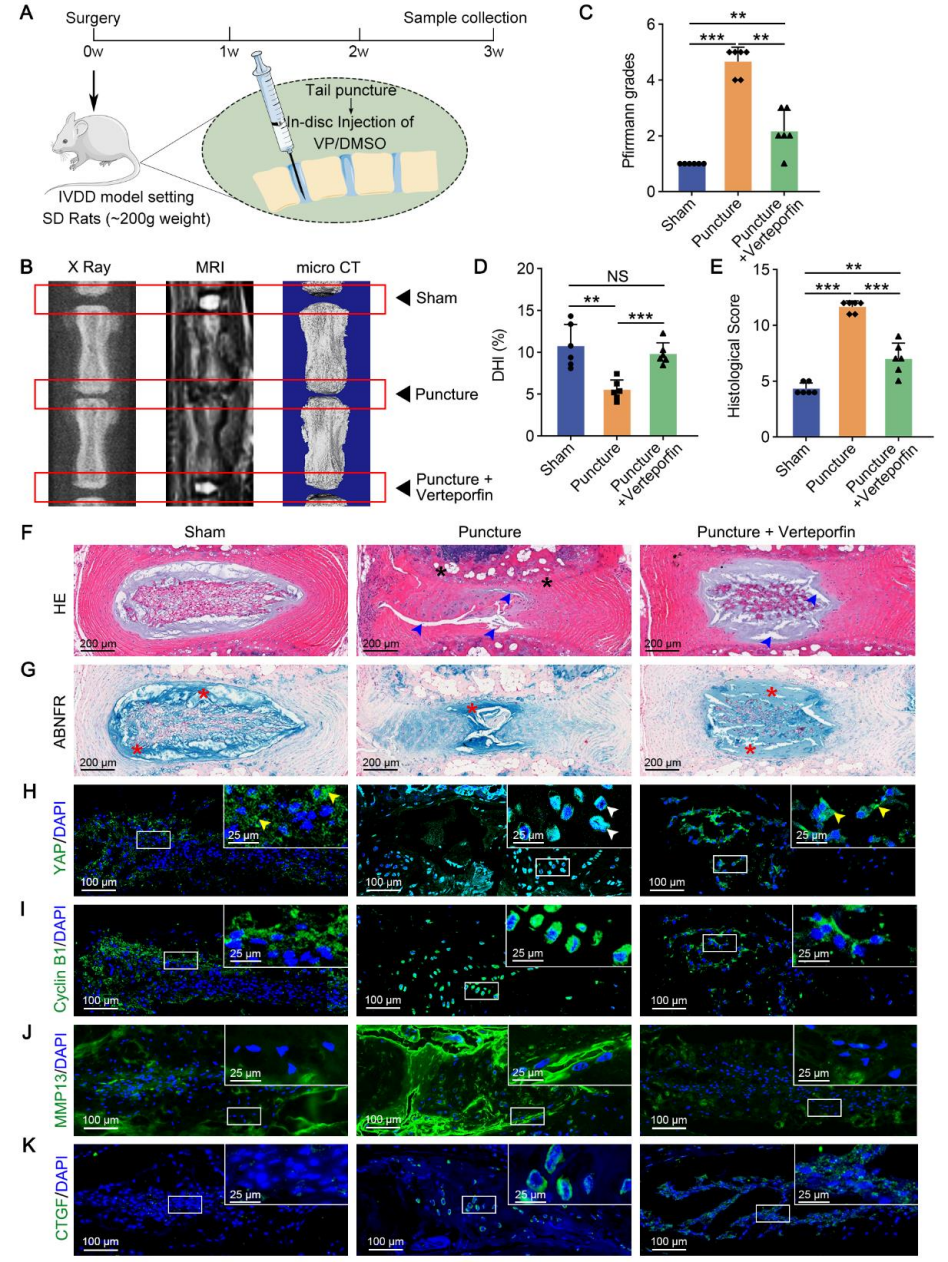
Verteporfin suppresses YAP/TEAD1-Cyclin B1 axis and alleviates IVDD. (A) Experimental design of disc needle puncture model and schematic of intradiscal injection of DMSO (0.25 ml/kg per disc) or VP (1.25 nmol/kg per disc). (B) X ray, MRI and micro-CT analysis of rat IVDs treated as in A. C) DHI of rat IVDs treated as in A based on micro-CT statistics (n=6).*p*-value was derived from One-way ANOVA followed by Tukey post hoc test. (D) Pfirrmann grades of rat IVDs treated as in A based on MRI signal density (n=6).*p*-value was derived from One-way ANOVA followed by Tukey post hoc test. E) Histological grading test of rat IVDs treated as in A (n=6).*p*-value was derived from One-way ANOVA followed by Tukey post hoc test. (F, G) HE and ABNFR staining of rat IVDs treated as in A. Fibrogenesis (black asterisk), gap between NP matrix (blue arrowheads) and proteoglycan (red asterisk) were labeled. (H-K) Fluorescence staining analysis of YAP, Cyclin B1, MMP13 and CTGF expression in rat IVDs treated as in A. Extranuclear (yellow arrowheads) and endonuclear YAP (white arrowheads) were labeled. Data are presented as the mean ± SD values. ^*^p < 0.05, ^**^p < 0.01, ^***^p < 0.001.

To further investigate the effects of YAP, we depleted YAP in rat NPCs seeded on rigid hydrogel. Decreased protein levels of YAP, p-YAP and TAZ were confirmed by western blots and fluorescence staining (*p* < 0.001 for YAP, *p* < 0.01 for p-YAP, *p* < 0.05 for TAZ, Fig. 4D, E; Supplementary Fig. 3A). However, TEAD1 expression was not altered, suggesting that YAP does not directly regulate TEAD1 production (*p* = 0.928, Fig. 4D, E). Through EdU staining, we found that YAP depletion suppressed rat NPCs proliferation with the positive rate in knockdown group nearly 50% lower than that in the control group (*p* < 0.01, Fig. 4F, G). Cell cycle analysis showed more NPCs arrested in G2/M phase after YAP knockdown (*p* <0.05, Fig. 4H). Moreover, downregulated Cyclin B1 and CDK1 and upregulated Cyclin D1 were found in rat NPCs with YAP ablation, indicating that Cyclin B1 and CDK1 are responsible for YAP-induced proliferation (*p* < 0.01 for Cyclin B1, *p* < 0.05 for CDK1, *p* < 0.05 for Cyclin D1, Fig. 4I, J; Supplementary Fig. 3B-E). Additionally, we examined the function of YAP in cell anabolism. YAP knockdown increased chondroid elements (ACAN and Collagen II) and decreased fibrotic elements (MMP13 and CTGF) in rat NPCs, reversing the degenerative phenotypes induced by elevated matrix stiffness (*p* < 0.01 for ACAN, *p* < 0.05 for Collagen II, *p* < 0.01 for MMP13, *p* < 0.05 for CTGF, Fig. 4K, L). In summary, activation of YAP is responsible for promoting the proliferation and fibrotic anabolism of NPCs.

### YAP/TEAD1 targets Cyclin B1 to induce changes in cell cycle and anabolic phenotypes

Endonuclear colocalization of YAP/TEAD1 was verified by fluorescence staining on rigid hydrogel (Fig. 5A), mediating downstream gene induction in rat NPCs[42]. To further validate whether TEAD1 regulates Cyclin B1 expression, we performed JASPAR database analysis and identified eight putative TEAD1 recognition motifs (TRMs) with over 85% confidence in the *CCNB1* promoter region (Fig. 5B). Chromatin immuno-precipitation assays of two deposited datasets (GSE41509 and GSE62275) showed an overlapping 406 bp peak of TEAD1 binding enrichment in the promoter of *CCNB1* between Chr5: 69,166,844-69,167,250, with a 92.2% confidence (Fig. 5C). Furthermore, a luciferase reporter plasmid containing the 2 kb proximal *CCNB1* promoter showed a positive response to TEAD1 overexpression (*p* < 0.01, Fig. 5D), suggesting that YAP/TEAD1 directly targeted and positively regulated *CCNB1*. We further examined whether TEAD1 and Cyclin B1 are involved in cell cycle and anabolism of NPCs. TEAD1 overexpression decreased the proportion of cells arrested in G2/M phase and *CCNB1* depletion increased the proportion (*p* < 0.001 for TEAD1 overexpression, *p* < 0.01 for *CCNB1* depletion, Fig. 5E, F). *CCNB1* ablation also augmented chondral production (ACAN and Collagen II) and suppressed fibrotic anabolism (MMP13) after TEAD1 overexpression in rat NPCs (*p* < 0.01 for ACAN, *p* < 0.01 for Collagen II, *p* < 0.05 for MMP13). However, expression of CTGF at the protein level was upregulated by TEAD1 but not altered by Cyclin B1, indicating another regulatory pathway (*p* = 0.231, Fig. 5G, H). Collectively, YAP/TEAD1 directly targets Cyclin B1 to induce cell proliferation and fibrotic phenotypes in rat NPCs.

### Fibro NPCs with high YAP expression enhance fibrogenesis in degenerated discs

We hypothesized that if a particular population of NPCs with high YAP expression is present in human NP tissues, it might be identifiable in data generated from previous studies. The scRNA-seq data of 39,732 human NPCs from a deposited dataset (GSE165722) were analyzed[43]. Cells were segregated into hypertrophic chondrocyte-like NPCs (HTCL NPCs), effector NPCs, homeostatic NPCs, regulatory NPCs, fibro NPCs and adhesion NPCs (Fig. 6A). Two clusters of human NPCs were identified as being responsible for ECM synthesis. Briefly, HTCL NPCs highly expressed chondrogenic genes such as *ACAN* and *COL2A1*, while fibro NPCs expressed a distinct set of genes promoting fibrogenesis such as*COL1A* and *MMPs* (Fig. 6B). All cells were further divided into two groups (mild and severe) based on the grade of degeneration. GO analysis verified the enrichment of the Hippo-YAP/TAZ signaling pathway in the severe group (Fig. 6C). Importantly, we confirmed elevated expression of both YAP- and anabolism-related genes in fibro NPCs (Figs. 6D, E). The proportion of fibro NPCs increased gradually with the grades of degeneration, indicating aberrant proliferation (Fig. 6F). Elevated expression of *TEAD1* and *CCNB1* was also found in degenerative procedures. Of note, *YAP* expression in total NPCs increased in grade III and decreased sharply in grade IV, indicating that YAP functions mostly in the early stage of IVDD (Fig. 6G). These data suggest that proliferation of high-YAP fibro NPCs contributes to elevated fibrogenesis during disc degeneration.

### Verteporfin suppresses YAP/TEAD1-Cyclin B1 axis and alleviates IVDD

To identify how YAP functions in the IVDD model, we screened out two small molecular drugs restraining YAP/TEAD1-Cyclin B1 axis, verteporfin and nocodazole. Verteporfin acts as an inhibitor of YAP/TEAD interaction [44], and nocodazole is a synthetic tubulin-binding agent that depolymerize microtubules to intercept mechanical signal transduction [45, 46]. Cell cycle analysis showed suppressed proliferation characterized by an increased proportion of G2/M phase in both groups (*p* < 0.001 for verteporfin,*p* < 0.05 for nocodazole, Supplementary Fig. 4A, B). Moreover, verteporfin treatment suppressed YAP and Cyclin B1 expression and enhanced chondral anabolism including ACAN and Collagen II (*p* < 0.05 for YAP, *p* < 0.05 for Cyclin B1, *p* < 0.01 for ACAN, *p* < 0.01 for Collagen II, Supplementary Fig. 4C, D). However, nocodazole treatment resulted in an upregulation of CTGF and no difference in ACAN protein levels (*p* < 0.001 for CTGF, *p* = 0.721 for ACAN, Supplementary Figs. 4C, D). Therefore, verteporfin showed inferior efficacy to nocodazole in ameliorating degeneration and was intradiscally injected for IVDD treatment in a needle puncture model (Fig. 7A). MRI results showed degradation of ECM in puncture group, characterized by lower signal density than control group (Fig. 7B). Meanwhile, the height of intervertebral space decreased significantly after needle puncture. (Fig. 7B). In puncture plus verteporfin group, both increased disc height index (DHI) and reduced Pfirrmann grades revealed that injection of verteporfin maintained the structure and water content of discs (Figs. 7C, D). Degenerative morphology, including enhanced fibrosis and an enlarged gap within NP tissues, was observed after acupuncture compared with the sham group (*p* < 0.001, Figs. 7E, F). According to ABNFR and fluorescence staining, punctured IVDs displayed lower proteoglycan (mainly ACAN) production, increased fibrosis (MMP13 and CTGF), and superior YAP and Cyclin B1 levels than those of the sham group (Figs. 7G-K). Importantly, injection of verteporfin suppressed YAP/TEAD1-Cyclin B1 axis and alleviated degenerative phenotypes, resulting in shrunken gap, restored proteoglycan and reduced fibrogenesis compared with those of the puncture group (Fig. 7G-K). Thus, verteporfin shows excellent curative effects for IVDD and emphasizes that YAP signaling pathway accelerates NP degeneration.

**Figure 8.**
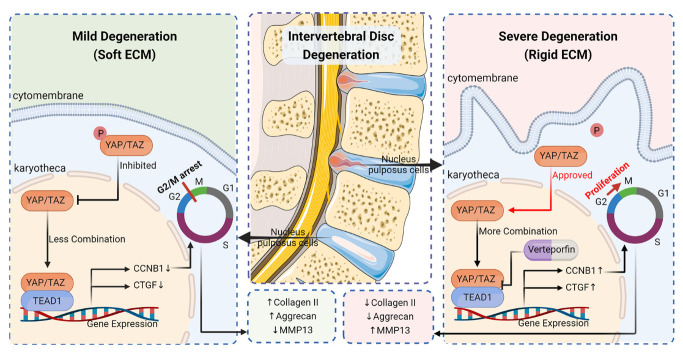
Schematic illustration showing the possible mechanism by which YAP induces NPCs proliferation and degenerative phenotypes. In severely degenerated NP tissues, nuclear translocation of YAP is promoted by increased matrix stiffness. Subsequently, YAP/TEAD1-Cyclin B1 axis promotes high-YAP fibro NPCs proliferation, leading to degenerative phenotypes in NP tissues.

## DISCUSSION

Matrix stiffness of NP tissues significantly increased during IVDD. Rigid matrix stimulates cell proliferation and promotes fibrotic phenotypes of NPCs, but the underlying molecular mechanism has remained elusive. Successful treatments may thus require an in-depth understanding of how matrix stiffness affects NPCs. This study showed that YAP/TEAD1 directly targets and positively regulates Cyclin B1 in NPCs on rigid hydrogel. Cyclin B1 induced high-YAP fibro NPCs proliferation, resulting in fibrogenesis. *CCNB1* depletion increased chondroid synthesis and reduced fibrotic anabolism of NPCs *in vitro*. Inhibition of YAP/TEAD interaction by verteporfin suppressed cell proliferation and alleviated degeneration caused by needle puncture *in vivo*. Together, our data provided critical insights into the correlation between matrix stiffness and YAP/TEAD1-Cyclin B1 axis in NP tissues, indicating a therapeutic target for IVDD (Fig. 8).

YAP is an important effector of mechanical signals generated from cell-cell contacts and ECM interactions [38]. Rigid matrix activates YAP in various pathological processes such as breast cancer development [22] and renal fibrogenesis [47]. Consistent with previous studies [11], we found an obvious augmentation of YAP expression in early stage of degeneration (Pfirrmann grade III). However, YAP dramatically decreased in NPCs of Pfirrmann grade IV, according to scRNA-seq analysis. Likewise, the density of NPCs shows a rapid decline in Pfirrmann grade IV [48]. Cell-cell contact is suppressed by reduced density and thus responsible for YAP downregulation in advanced stage of IVDD [49]. These results suggest that YAP activation occurs mainly in the early stage of NP degeneration, emphasizing the importance of early intervention for IVDD therapy.

Stimulated proliferation on rigid substrate is accompanied by changes in cell cycle, which is regulated by various Cyclin proteins [50]. For example, Cyclin D controls the restriction point in G1 phase, and Cyclin B is involved in the G2/M checkpoint [39, 51, 52]. In mouse embryo fibroblasts cultured on stiffer substrates, Cyclin D1 and Cyclin A increased significantly, with more cells in S phase [53]. In A549 and MDA-MB-231 cells, Cyclin D1 is considered a critical protein that promotes proliferation on rigid hydrogel [54, 55]. YAP/TEAD1 has been proven to interact with the promoter region of *CCND1* to induce its transcription [56]. However, we observed no obvious upregulation of Cyclin D1 in rat NPCs seeded on rigid hydrogel, whereas we found enhanced Cyclin B1 expression and conjunction between TEAD1 and *CCNB1* promoter region. Expression of Cyclin proteins differs in cell types, which is especially obvious in the case of meiotic cyclins (Cyclin A and B) or G1-type cyclins (Cyclin D and E) [57, 58]. For instance, Cyclin D and Cyclin B are both highly expressed in mouse embryonic fibroblasts. However, mouse embryonic stem cells can proliferate in the absence of D- and E-type cyclins [59]. Thus, it is plausible that YAP/TEAD1 promotes NPCs proliferation mainly through Cyclin B1 rather than Cyclin D1 due to the specificity of cell types.

Activated YAP/TEAD1 leads to degenerative phenotypes of NPCs [60-63]. A chondral subcluster highly expressing fibrotic genes, including *COL1A1*, *MMP13* and *FOSL1*, has been identified in human NPCs and only exists in degenerated tissues [64, 65]. Fibroblast proliferation has been confirmed [29], and the proportion of fibro-chondrocytes gradually increases with the progression of degeneration [66]. Through analysis of a deposited scRNA-seq database, we identified one cluster of human NPCs highly expressing both YAP- and fibrosis-related genes, named fibro NPCs. The proportion of high-YAP fibro NPCs increased significantly with the grade of degeneration. Therefore, fibro NPCs with high YAP expression are critical for fibrogenesis during IVDD.

Moreover, Cyclin proteins were reported to induce differentiation of various stem or progenitor cells. In cortical progenitors, Cyclin B is one of the strongest transcriptional signatures defining multipotent RGCs, and lineage-committed progenitors are enriched in Cyclin D [67]. During erythroid differentiation, lineage-specific changes include the sustained upregulation of Cyclin A and Cyclin D1 expression, blocking Cyclin D results in the inhibition of lineage commitment [68]. NPCs is a general term for all cells in NP tissues, which is diversified including NP progenitors, transient NPCs, regulatory NPCs and homeostatic NPCs according to previous work [69]. Existence of progenitors in NPCs is further verified by other researchers [70, 71]. Of note, fibro NPCs is found at the start of the pseudospace trajectory through monocle analysis [43], thus it is plausible that rigid matrix elevated Cyclin B1 in fibro NPCs to maintain its phenotypes and suppressed Cyclin D1 to inhibit its differentiation into HTCL NPCs. Further studies are required to confirm the existence of fibro NPCs *in vivo*.

Although our study provided detailed insights into YAP-induced proliferation on stiffer substrates, there are potential limitations. First, rat NPCs were used for *in vitro* experiments. Even though we identified overexpression of YAP and Cyclin B1 in human NP tissues through fluorescence staining and scRNA-seq analysis, human NPCs should be used to verify the existence of YAP/TEAD1-Cyclin B1 axis. Second, the hydrogel substrates only mimicked the two-dimensional environment of NPCs in tissues, lacking the three-dimensional influence of mechanical stimulation. Nevertheless, our data strongly indicate that fibro NPCs proliferation induced by YAP activation accelerates fibrogenesis during degeneration. YAP/TEAD1-Cyclin B1 axis may be a promising novel therapeutic target for IVDD.

## Supplementary Materials

The Supplementary data can be found online at: www.aginganddisease.org/EN/10.14336/AD.2023.00205-1. Processed scRNA-seq data (gene counts per cell) were downloaded from Tu et al., 2021 (GSE165722).


