# Supplementary material for: Matrix Stiffness Activating YAP/TEAD1-Cyclin B1 in Nucleus Pulposus Cells Promotes Intervertebral Disc Degeneration
Source: Aging Dis. 2023 Oct 1;14(5):1739–56. doi: 10.14336/AD.2023.00205-1 (PMC10529754; doi:10.14336/AD.2023.00205-1)
Supplement: Supplementary file 1 [file AD-14-5-1739-s.pdf]

SUPPLEMENTARY DATA

Matrix Stiffness Activating YAP/TEAD1-Cyclin B1 in Nucleus Pulposus Cells Promotes Intervertebral Disc Degeneration

Zijie Zhou[#], Yinxuan Suo[#], Jinyu Bai[#], Fanguo Lin, Xiang Gao, Huajian Shan, Yichao Ni, Xiaozhong Zhou[*], Lei Sheng[*], Jun Dai[*]

© 2022. Zhou Z et al. Published online at http://www.aginganddisease.org/EN/10.14336/AD.2023.00205-1

SUPPLEMENTARY DATA

Supplementary Table 1. Composition of polyacrylamide hydrogels

Components	Soft	Rigid
Acrylamide (40%) (mL)	0.75	2.5
Bis-acrylamide (2%) (mL)	1.5	1.5
Water (mL)	7.75	6

Supplementary Table 2. Patient demographics.

Case number	Gender	Age (years)	Pfirrmann grade	Diagnosis
Case 1	male	26	II	Lumbar disc herniation
Case 2	male	31	II	Lumbar disc herniation
Case 3	female	34	II	Lumbar disc herniation
Case 4	female	57	II	Lumbar spinal stenosis
Case 5	male	15	III	Lumbar disc herniation
Case 6	male	26	III	Lumbar disc herniation
Case 7	female	52	IV	Lumbar spinal stenosis
Case 8	female	54	IV	Lumbar disc herniation
Case 9	female	57	IV	Lumbar disc herniation
Case 10	male	61	IV	Lumbar disc herniation
Case 11	female	33	V	Lumbar disc herniation
Case 12	female	33	V	Lumbar disc herniation

Supplementary Table 3. Sequences of siRNAs and plasmids

Name		Sequence
YAP siRNA	Sense:	5’-GCCAUGAACCAGAGGAUCATT-3’
	Anti-sense:	5’-UGAUCCUCUGGUUCAUGGCTT-3’
CCNB1 siRNA	Sense:	5’-GGCUAAUACAGGUUCAGAUTT-3’
	Anti-sense:	5’-AUCUGAACCUGUAUUAGCCTT-3’
Negative control	Sense:	5’-UUCUCCGAACGUGUCACGUTT-3’
	Anti-sense:	5’-ACGUGACACGUUCGGAGAATT-3’
TEAD1 plasmid	5’-ATTGAGCCCAGCAGCTGGAGCGGCAGTGAGAGCCCTGCC<br>GAAAACATGGAAAGGATGAGTGACTCTGCAGATAAGCCAATTGACAATGATGCAGAAGGGGTCTGGAG<br>CCCCGACATCGAGCAAAGCTTTCAGGAGGCCCTGGCTATCTATCCACCATGTGGGAGGAGGAAAATCA<br>TCTTATCAGACGAAGGCAAAATGTATGGTAGGAATGAATTGATAGCCAGATACATCAAACTCAGGACA<br>GGCAAGACGAGGACCAGAAAACAGGTGTCTAGTCACATTCAGGTTCTTGCCAGAAGGAAATCTCGTGA<br>TTTTCATTCCAAGCTAAAGGATCAGACTGCAAAGGATAAGGCCCTGCAGCACATGGCGGCCATGTCCTC<br>AGCCCAGATCGTCTCGGCCACTGCCATTCATAACAAGCTGGGGCTGCCTGGGATTCCACGCCCGACCTT<br>CCCAGGGGCGCCGGGGTTCTGGCCGGGAATGATTCAAACAGGGCAGCCAGGATCCTCACAAGACGTCA<br>AGCCTTTTGTGCAGCAGGCCTACCCCATCCAGCCAGCGGTCACAGCCCCCATTCCAGGGTTTGAGCCTG<br>CATCGGCCCCAGCTCCCTCAGTCCCTGCCTGGCAAGGTCGCTCCATTGGCACAACCAAGCTTCGCCTGG<br>TGGAATTTTCAGCTTTTCTCGAGCAGCAGCGAGACCCAGACTCGTACAACAAACACCTCTTCGTGCACA<br>TTGGGCATGCCAACCATTCTTACAGTGACCCATTGCTTGAATCAGTGGACATTCGTCAGATTTATGACA<br>AATTTCCTGAAAAGAAAGGTGGCTTAAAGGAACTGTTTGGAAAGGGCCCTCAAAATGCCTTCTTCCTCG<br>TAAAATTCTGGGCTGATTTAAACTGCAATATTCAAGATGATGCTGGGGCTTTTTATGGTGTAACCAGTC<br>AGTACGAGAGTTCTGAAAATATGACAGTCACCTGTTCCACCAAAGTTTGCTCCTTTGGGAAGCAAGTAG<br>TAGAAAAAGTAGAGACGGAGTATGCAAGGTTTGAGAATGGCCGATTTGTATACCGAATAAACCGCTCC<br>CCAATGTGTGAATATATGATCAACTTCATCCACAAGCTCAAACACTTACCAGAGAAATATATGATGAAC	

© 2022. Zhou Z et al. Published online at http://www.aginganddisease.org/EN/10.14336/AD.2023.00205-1

SUPPLEMENTARY DATA

	AGTGTTTTGGAAAACTTCACAATTTTATTGGTGGTAACAAACAGGGATACACAAGAAACTCTACTCTGC<br>ATGGCCTGTGTGTTTGAAGTTTCAAATAGTGAACACGGAGCACAACATCATATTTACAGGCTTGTAAAG<br>GACTGA-3’
CCNB1 promoter-luciferase plasmid	5’-TTGAGTAAAAGAAACCATTCTGACCGCCAGGTGCAGTGG<br>CTCACGCCTTCAATACCAGCACTTTGGGAGGTTGAGGCGGGTGGACATTATACGTCATTATATATTTGTC<br>AAAACCCGTAGAAGACACAACACCTAGAGTGAACCTTAATGTAAACTATGGACTTTGGGTAATACTGT<br>GGTGTCAATGTAGGTTAACAGATTATAATTAAAGTACAACTTTAATGTGGGGTGTCAATAGTGGGGAAG<br>GCAGGTGAAATGCTCTATACTTTCTGCTAAGTTTTGCTGCGAACCTAAAACTGCTCTAAAAAATAAAGC<br>ATATAAAAGGAAAAATTTGGCCGGGCGTGGTGGCTCACGCCTGTAATCGCAGCACTTTGGGAGGCCGA<br>GGCGGGCGGATCACCAAGTCAGGAGATCGAGACCATCCTGGCTAACATGGTGAAACCCCGTCTCTACC<br>AAAAATACAAAAAATTAGCCGGCCGTTGTGGCGGGCGCCTGCCGTCCCAGCTACTCGGGAGGCTGAGG<br>CAGGAGAATGGAGTGAACCCTGGAGGTGGAGGTTGCAGTGAGTCGAGATCGCACCACTGCACTCCAGC<br>CTGGGCGACAGATCGAGACTCCGTCTCAAAAAAAAGTAAAAAAAAAAATTTTTAAACTGGAACACAGA<br>GGATAAGTAATCCTGCTTCGCCCCTTGCCTCTCGCCCCTGCATGGGGCGAGGAAGGATTGATCAAACCC<br>AGAAAGACTGAGTGAGAATGGATGTTGAACACAGAACTGAAGGATGAATGGGAGTTACCTGAATAGA<br>ACCCAGGGGCTTGAATGCAAGAAGAGGCGGGCATTCCAGGCAGAGGAGAGCAAGGGTAAGGGCCCCA<br>CGGGAGGCATTCGAGTAGGAGGGTGAACTATGATGACAGAAGACTCAATAACGATCCAAAGAAACCA<br>AATGATTGGGCGCCTTCTTTCGGATCCGTGACTTCCAGCGCCAGGAGTCTCTATTGGCTCTTATACCGTT<br>GCTCTATGGGATAGCAATGTTTTTGTCCTTCAGCCTCCCCTCCAATTGCTGAGCTGCTGGTGTGTTTTGA<br>GGAGTAGAAGGCAAAAAGAACCCTCTTGTTTTTCTTTGGATCTAGAGAGAATCTGAGCAAATGACAAA<br>GCAAATGGGGTAAAATGTCTTTTTGTTTAGTTTTTCCTGATTTTCCCATGAGAGGCAAATACATGTTAAG<br>GATAGTTGAATCTGAGTAAAGGGCATAGAAATATTCCTTACAAGATTTTTGTTGGCAACTGGTCTAAGT<br>ATGAAATTATTTCCAAATAGAAAGCTAAAACAAACAAAACAATTGGCCTTGGGAAACTGGACAATCTT<br>GAAGTAATCAAGTAATATTGTTAAGGGACAATCAGTGTTGTGAAAACAACACGGATACACCCCCTCCCT<br>CCCCCTCAAAAAAAAAAACCCTAAATTCAGTTCCCCCGTTGCTAATGTGTGACCCTGGCAAAGTCATCT<br>AAGTCGCTGAGCTTCAGTTCCTCCAACCCAGAGAGTTGTTGCAACGATCAAATGAAAGAATGTCTATTA<br>AAGCCTTTCATGAACTATATTATTGCTGCTACCGTAGAAATGGAAAGTGTGCAACACTAGATCCAAAAC<br>TACTTTTGACACTTCTGAGACTGTGGCCGCGCCTCTGTCACCTTCCAAAGGCCACTAGGCCTTTCCTGAG<br>CTGGCATTGGCAACGCACACTCTTGCCCGGCTAACCTTTCCAGGTGGGCGGCGCACTGGCTTCACTGCT<br>CTCCAGGTGGCCGCTGCAGCTGCCCGAGAGCGCAGGCGCAGAGGCAGACCACGTGAGAGCCTGGCCAG<br>GCCTTCCGGCCTAGCCTCACTGTGGCCCCGCCCCTCTCGAACGCCTTCGCGCGATCGCCCTGGAAACGC<br>ATTCTCTGCGACCGGCAGCCGCCAATGGGAAGGGAGTGAGTGCCACGAACAGGCCAATAAGGAGGGA<br>GCAGTGCGGGGTTTAAATCTGAGGCTAGGCTGGCTCTTCTCGGCGTGCTGCGGCGGAACGGCTGTTGGT<br>TTCTGCTGGGTGTAGGTCCTTGGCTGGTCGGGCCTCCGGTGTTCTGCTTCTCCCCGCTGAGCTGCTGCCT<br>GGT-3’

Supplementary Table 4. Primer sequence

Primer		Sequence
Ccnb1	F:	5’-TAGACACCAACTCTGCAGCA-3’
	R:	5’-GCTGGCTTTGATCTCAGACG-3’
Ccnd1	F:	5’-GATGCTAGAGGTCTGCGAGGA-3’
	R:	5’-CGGCTCTTCTTCAGGGGCTC-3’
Cdc25a	F:	5’-CGCCGCCTGCTCTTCACTTG-3’
	R:	5’-ATAGTCACTGCCCAGCCCTTCC-3’
Cdc25b	F:	5’-AACGGAGCAGGAGGGAACTCTG-3’
	R:	5’-TGAGGTAAGGATGGTGGCAGGAC-3’
Cdc25c	F:	5’-GCCAGAGCAAAGCCCAGGAAG-3’
	R:	5’-GTGTCTTCACTGCCACCGTCATAG-3’
Cdk1	F:	5’-AGGGCGCCGTGTATGTAATA-3’
	R:	5’-ACCTGTCCTACGAACATGCA-3’
Cdk4	F:	5’-GGCTGATGGATGTCTGTGCTACTTC-3’
	R:	5’-TGTCCGTAGGTCCTGGTCTATATGC-3’

© 2022. Zhou Z et al. Published online at http://www.aginganddisease.org/EN/10.14336/AD.2023.00205-1

Taz	F:	5’-ATTGGACGGCTGATTGCTGAGTG-3’
	R:	5’-GTAGGGTGGACTGTTAGGGAGGAC-3’
Tead1	F:	5’-CCCATCCCCAACACTCTTCT-3’
	R:	5’-TGTAAATGGCCCCAGTAGCA-3’
Yap	F:	5’-CCACTGTTGCTCTGCTCTTCTCTG-3’
	R:	5’-GGGCTCTGGCTATTTGTGGTTCTG-3’

Supplementary Table 5. Grading for morphology

		**Morphology changes under optical microscope**	**Grade**
I	Annulus fibrosus (AF)	Normal texture and free of damage and distortion	1
		The damaged and distortion area is less than 30%	2
		The damaged and distortion area is more than 30%	3
II	Boundary between AF and NP	Normal	1
		Micro disrupted	2
		Medium or severe disrupted	3
III	NP cells	Normal cells with large amounts of vacuoles	1
		Cells and vacuoles decreased slightly	2
		Cells decreased moderately or severely without vacuoles	3
IV	NP matrix	Normal gel appearance	1
		Slightly congealed	2
		Moderate or severe condensation	3

Supplementary Table 6. Differentially expressed genes (Soft vs Rigid, padj > 0.05)

Gene ID	log2FoldChange	padj
Rasd1	3.219613	9.27E-08
Ankle1	-3.1828	7.84E-07
Pimreg	-2.2476	7.52E-06
Ggt1	2.396	1.33E-05
Aurkb	-2.26089	6.43E-05
Yap1	-2.28711	0.000204
Hmgb2l1	-2.20864	0.000204
Cenpa	-2.0921	0.001505
Ckap2	-2.37405	0.00248
Isg20	1.977007	0.00257
Spag5	-2.32101	0.003121
Slc25a25	1.603557	0.003251
Plk1	-2.03559	0.003251
Tead1	-2.65133	0.003408
Fam111a	-4.59025	0.003408
Kif20b	-3.648	0.003705
Melk	-2.80341	0.004671
Pla2g4a	-2.97284	0.004751
Nusap1	-2.50038	0.004751
Lmcd1	2.485345	0.004751
Espl1	-2.47657	0.004751
Fbxo5	-2.17806	0.004751
Tpx2	-2.59382	0.005143
Ciart	1.628138	0.007211
Gins1	-2.30586	0.01104

© 2022. Zhou Z et al. Published online at http://www.aginganddisease.org/EN/10.14336/AD.2023.00205-1

Troap	-2.0385	0.011255
Pclaf	-2.39432	0.011869
Aunip	-2.62692	0.01251
AABR07066944.1	-2.57342	0.013575
Kif2c	-2.66102	0.013624
Traip	-2.20531	0.013624
Ppp2r2c	-6.48215	0.013624
Ccnb1	-2.80995	0.013624
Glb1l2	-3.68105	0.015984
Xkr5	-2.65825	0.0197
Slc2a4	2.419926	0.020674
LOC100359539	-2.81881	0.02406
Pgf	2.980391	0.026643
Cep72	-3.1864	0.026643
Kcnk4	2.650661	0.026643
Erfe	-4.88798	0.026643
Mad2l1	-1.96189	0.028214
Ube2c	-1.95218	0.030526
Hist2h4a	2.877234	0.030526
Cgnl1	-2.20258	0.030526
Ndc80	-2.78164	0.030721
Cenpe	-3.17282	0.032023
Taz	-2.18698	0.034086
Nuf2	-2.92912	0.034086
Ampd1	4.102278	0.034086
Cenpk	-2.1116	0.034086
Kif15	-2.48156	0.034086
Rem2	1.975882	0.034942
Gbp6	-6.16415	0.035226
Iqgap3	-2.37919	0.039585
Top2a	-3.06877	0.039585
Kif23	-2.7837	0.044693
Rgs20	-5.09955	0.04623
Ypel4	2.492485	0.046649
Htra3	1.404279	0.047239
Kif4a	-2.65892	0.04895

© 2022. Zhou Z et al. Published online at http://www.aginganddisease.org/EN/10.14336/AD.2023.00205-1

SUPPLEMENTARY DATA

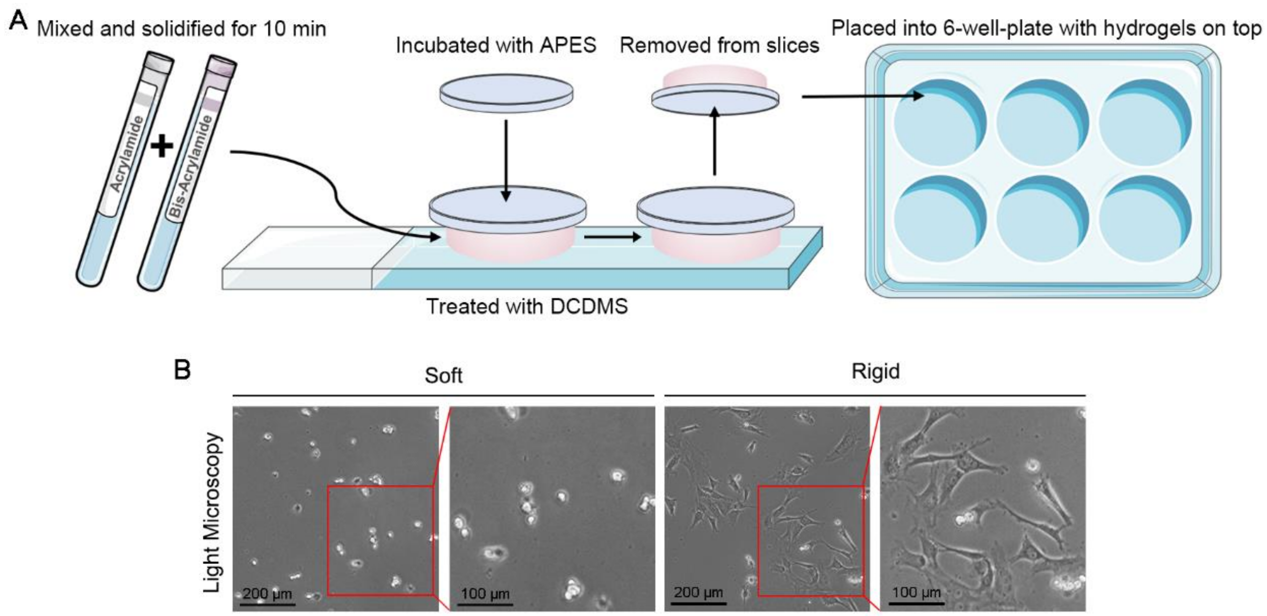

Supplemental Figure 1. Matrix stiffness of NP tissues is increased on degenerated discs. A) Experimental design for establishing polyacrylamide hydrogel substrates used for cell culture. APES 3-aminopropyltrimethoxysilane, DCDMS dichlorodimethylsilane. **B)** Morphology of rat NPCs on soft and rigid substrates under light microscopy.

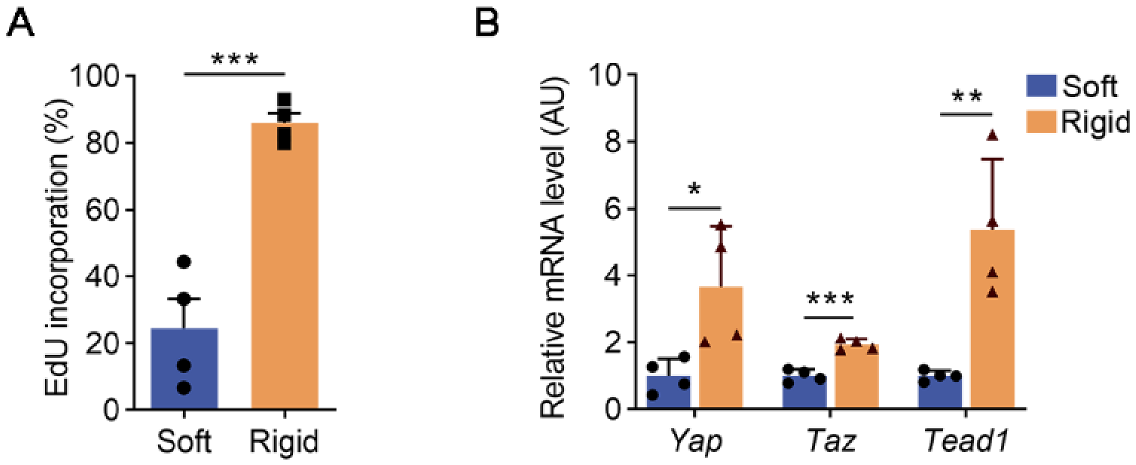

Supplemental Figure 2. Rigid hydrogel induces NPCs proliferation and YAP activation. A) Cell proliferation capacity analysis on rat NPCs on soft and rigid hydrogels through EdU staining (n=4). *p*-value was derived from Wilcoxon rank-sum test. **B)** Relative mRNA levels of *Yap* (n=4), *Taz* (n=4) and *Tead1* (n=4) in rat NPCs on soft and rigid hydrogels by qRT-PCR. *p*-value was derived from Wilcoxon rank-sum test. *Gapdh* was used as the loading control and results were relative to the soft group. Data are presented as the mean ± SD values. $^{*}p < 0.05$, $^{**}p < 0.01$, $^{***}p < 0.001$.

SUPPLEMENTARY DATA

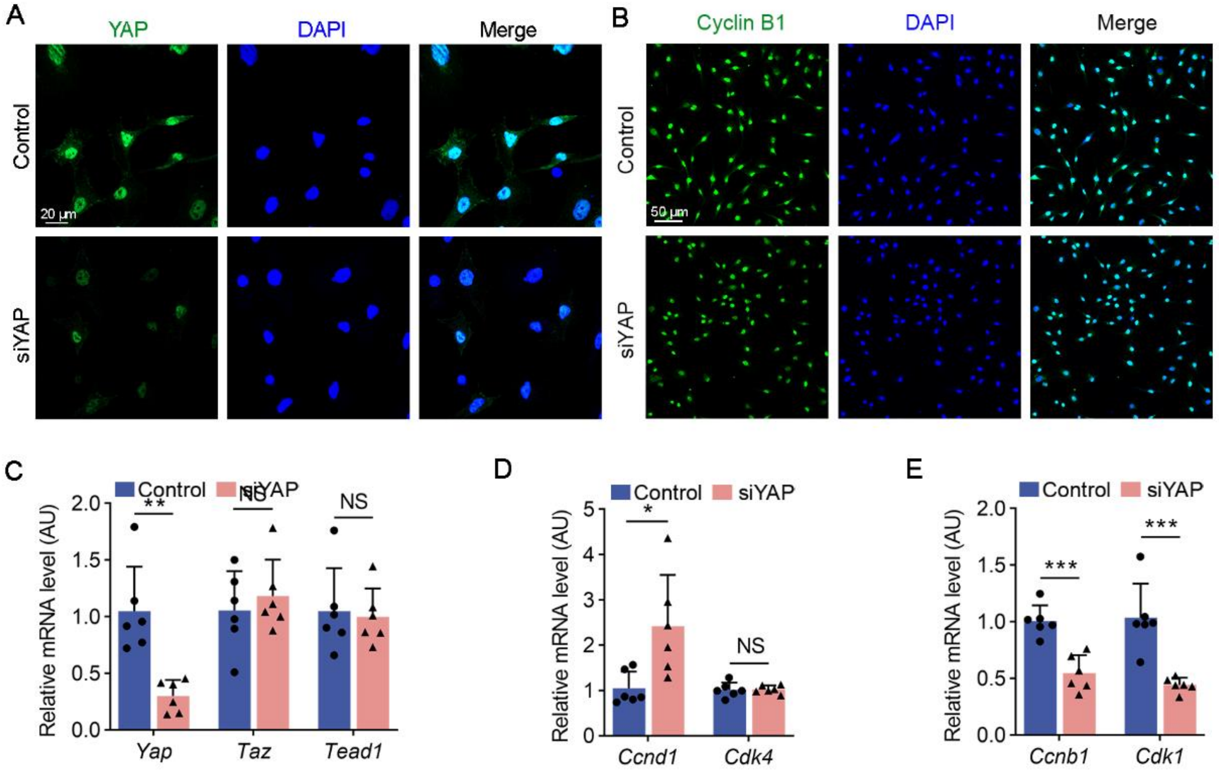

Supplemental Fig. 3 YAP knockdown disturbs cell cycle-related genes. A, B) Fluorescence staining analysis of YAP and Cyclin B1 expression in rat NPCs planted on soft and rigid hydrogels for 2 days. Nuclei were stained with DAPI. **C-E)** Relative mRNA levels of *Yap* (n=6), *Taz* (n=6), *Tead1* (n=6), *Ccnd1* (n=6), *Cdk4* (n=6), *Ccnb1* (n=6) and *Cdk1* (n=6) in rat NPCs transfected with siNC or siYAP (both were planted on rigid hydrogel) for 2 days. *p*-value was derived from two-tailed unpaired Student's t-test. *Gapdh* was used as the loading control and results were relative to the control group. Data are presented as the mean ± SD values. $^{*}p < 0.05$, $^{**}p < 0.01$, $^{***}p < 0.001$.

© 2022. Zhou Z et al. Published online at http://www.aginganddisease.org/EN/10.14336/AD.2023.00205-1

SUPPLEMENTARY DATA

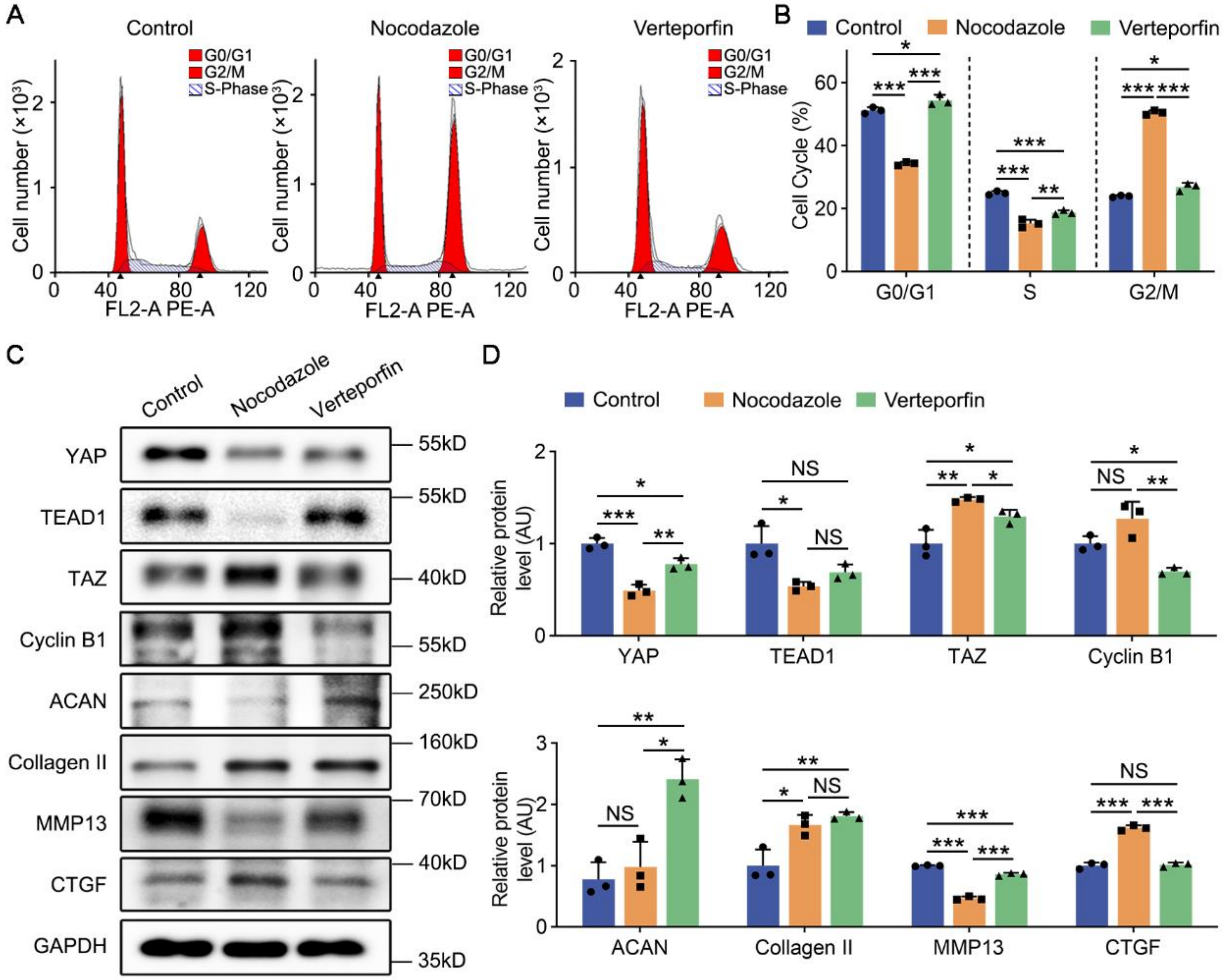

Supplemental Figure 4. Verteporfin suppresses YAP/TEAD1-Cyclin B1 axis and alleviates IVDD. A, B) Cell cycle analysis of rat NPCs treated with 0.25 µmol/L verteporfin or 5 µg/ml nocodazole for 2 days (n=3). *p*-value was derived from Kruskal-Wallis test. **C, D)** Western blot analysis of YAP (n=3), TEAD1 (n=3), TAZ (n=3), Cyclin B1 (n=3), ACAN (n=3), Collagen II (n=3), MMP13 (n=3) and CTGF (n=3) in rat NPCs treated as in **A**. *p*-value was derived from Kruskal-Wallis test. GAPDH was used as the loading control and results were relative to the control group. Data are presented as the mean ± SD values. $^{*}p < 0.05$, $^{**}p < 0.01$, $^{***}p < 0.001$.

© 2022. Zhou Z et al. Published online at http://www.aginganddisease.org/EN/10.14336/AD.2023.00205-1
